# Can real-time cmr provide adequate morphologic or functional data? A real-time CMR/SSFP comparison trial

**DOI:** 10.1186/1532-429X-13-S1-P7

**Published:** 2011-02-02

**Authors:** Wyatt D Unger, Maggie Diller, James D Dewar, Vincent L Sorrell

**Affiliations:** 1 University of Arizona, Tucson, AZ, USA

## Introduction

The objective of this trial was to assess the accuracy of cardiac magnetic resonance (CMR) images obtained via real-time CMR (RT-CMR) (GE: MR Echo) imaging sequences in comparison to steady-state free precession (SSFP).

## Purpose

Diagnostically useful cardiac MR images are currently obtained using SSFP imaging sequences. Longer acquisition times and breath holding make this sequence unsuitable for use in certain patient populations (eg. - children and sick adults). Unfortunately, RT-CMR images have lower spatial and temporal resolution than those obtained using SSFP sequences and are not currently used clinically.

## Methods

Forty nine consecutive patients (table 1) underwent both SSFP and RT-CMR images. Clinically relevant cardiac parameters (table 2) were measured in both diastole and systole utilizing SSFP and RT-CMR sequences. Measurements were made by considering the largest ventricular size to be diastole and the smallest to be systole. Measurements were made using common, consistent anatomic locations in both methods, were performed in random order, and were blinded to the results from the other sequence. Cardiac dimensions measured in each imaging modality were then compared using a paired t-test as well as a Passing and Bablock regression analysis.

**Table 1:** Baseline Characteristics of the Study Population

Number of patients	49
	Mean

Age, yrs	53 + 21 (8-93)
Female Gender	31
BSA, M2	1.9 + 0.19 (1.6-2.3)
LV Diastolic Area	3741.57±1013.63 (2384.91-7886.43)
LV Systolic Area	2166.37+1088.42 (994.70-6442.82)

**Table 2:** Summary of data from the paired t-test, and Passing and Bablock regression analysis

Measurement	Paired t-test p value	Intercept	95% CI	Slope	95% CI
LV Diastolic Area (mm^2^)	0.0470	-175.39	-662.23 to 207.50	1.0538	0.95 to 1.20
LV Systolic Area (mm^2^)	0.0801	-843.79	-2527.17 to 28.74	2.5065	1.76 to 4.06
FAC (%)	0.4729	-0.0060	-0.068 to 0.054	0.9966	0.84 to 1.18
Aortic Root (mm)	0.5791	1.8565	-15.82 to 3.26	0.9323	0.88 to 1.51
LA Area Systole (mm^2^)	0.5448	-3.6121	-278.13 to 244.86	0.9966	0.89 to 1.14
RV Diastolic Area (mm^2^)	0.1434	-496.57	-1268.73 to 334.27	1.2269	0.91 to 1.56

## Results

**Figure 1:**
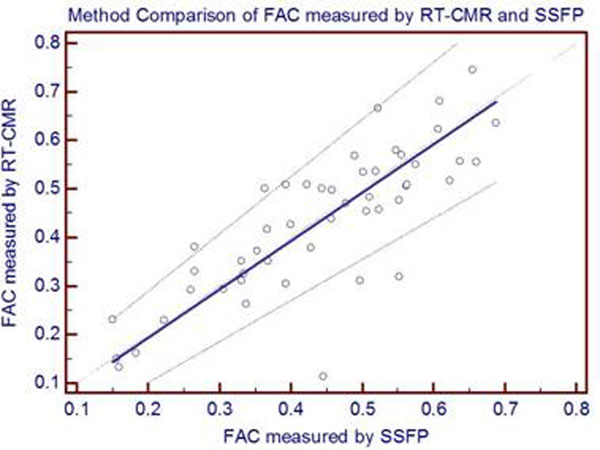
Plot of a Passing and Bablock regression of LVFAC measured by RT-CMR vs. SSFP. The regression equation is y=-0.005980 + 0.9966x and the Cusum test for linearity P.0.1 shows no significant deviation from linearity.

**Figure 2:**
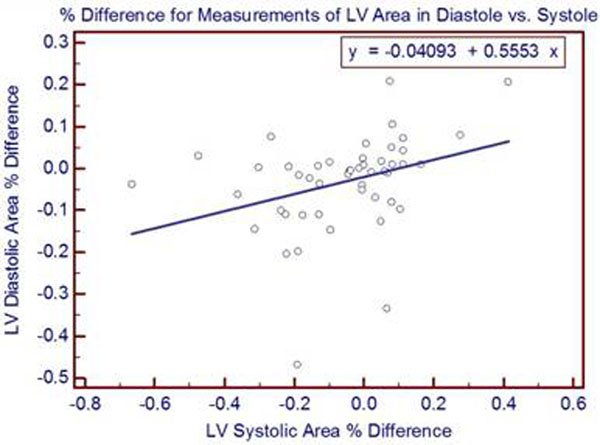
A regression of % difference in measured values between SSFP and RT-CMR between LV diastolic area and LV systolic area. The intercept = -0.041 (P=0.14) and the slope =0.553 (P=0.019), with an analysis of variance significance level of P=0.019.

With the exception of LVEDA, our data show no statistical difference between the measurements obtained using either CMR sequence (figures 1 and 2). The slope and intercept for a Passing and Bablock regression are shown below and demonstrate no significant deviation from linearity (ALL P VALUES N >0.10). RT-CMR exhibits a consistent, though not statistically significant underestimation of LV areas in both systole and diastole. Furthermore, analysis of the relationship between the percentage difference of these methods showed this difference to be highly, significantly correlated.

## Conclusions

RT-CMR provides clinically useful morphologic and functional LV and RV data compared to SSFP imaging. In this highly variable clinical population of pediatric and adults patients referred for CMR studies, there was no significant difference between SSFP and RT-CMR measurements with the exception of LV diastolic area.

